# Predictors of work-related musculoskeletal symptoms in shoulders among nursing assistants working in nursing homes

**DOI:** 10.7717/peerj.11152

**Published:** 2021-05-03

**Authors:** Kin Cheung, Ka Yan Ma, Hin Hei Cheung, Chun Ho Lee, In Mink Mavis Chan, Sin Ting Cheung, Wing Yee Chung, Sun Sun Yeung, Wing Chi Lo

**Affiliations:** School of Nursing, The Hong Kong Polytechnic University, Kowloon, Kowloon, Hong Kong

**Keywords:** Epidemiology, Shoulders, Musculoskeletal health, Body mass index, Workstyle

## Abstract

Nursing assistants (NAs) working in nursing homes (NHs) are at higher risk for work-related musculoskeletal symptoms (WRMSs) than their counterparts working in other health care settings. Worldwide, NAs have ranked shoulders in the top three body parts at risk of WRMSs. However, factors associated with their shoulder WRMSs are currently unknown. The aim of this study was to identify these associated risk factors among NAs working in NHs. 440 NAs from 47 nursing homes (with 60–90% response rate from each nursing home), recruited by convenience sampling, participated in this cross-sectional study in 2014–2015. A validated and reliable questionnaire was used for data collection. Information on demographic, job content questionnaire (JCQ), perceived physical exertion (PE), workstyle, ergonomic and manual handling knowledge and other work-related factors was collected using a self-administered questionnaire. 53% of the participants reported experiencing with WRMSs in their shoulders. Nine associated factors of shoulder WRMSs were identified using bivariate analysis. With the adjustment of age and gender using multivariable logistic regression, body mass index (OR = .931, 95% CI [.874–.991]), job title of health workers (OR = 2.72, 95% CI [1.18–6.25]) and workstyle-working through pain (OR = 1.06, 95% CI [1.01–1.11]) remained as predictors. Effort should be directed at integrating “workstyle intervention” into lifestyle physical activity training for NAs.

## Introduction

Globally, the increase in the ageing population is unprecedented. The estimated number of people aged 65 or above in 2050 is 1.5 billion, accounting for 16% of the world’s population; this is nearly triple the numbers in 2010 (World Health Organization, 2015). This continuous growth is posing multiple challenges to the healthcare system, especially to elderly care services. Demands for nursing home care services have increased drastically as the numbers of care-dependent seniors rises (World report on ageing and health, 2015). The projected number of nursing home places needed will have increased by 32.3% and 85.7% by 2025 and 2035 respectively in the United Kingdom (UK) (Kingston et al., 2017); and by 40% by 2030 in the United States (US) (Public Policy Institute of California, 2020).

To meet the demand for nursing home-care services, the number of nursing health-care workers will increase significantly as well. In nursing homes (NHs), nursing assistants (NAs) are the frontline workers providing the direct care to the elderly, including turning, lifting, changing clothes, changing incontinence pads and transferring them. All of these care-related activities pose musculoskeletal risks to NAs (Cheung et al., 2018). Due to the job nature and the demanding workload, NAs have been ranked at the top for work-related musculoskeletal symptoms (WRMSs), such as pain, discomfort and numbness (Luime et al., 2004), in hospitals, NHs (Meyer & Muntaner, 1999; Pelissier et al., 2014) or other health settings (Jensen, 1987; Myers, Silverstein & Nelson, 2002). In the US, 53% of the WRMSs cases were reported by NAs in 2013 (Bureau of Labor Statistics, 2016). Across countries, NAs have ranked shoulders in the top three body parts at risk of WRMSs. For example, shoulders were the body part experienced WRMSs the most frequently in Hong Kong (Cheung et al., 2018) and Korea (Smith et al., 2003a; Smith et al., 2003b), and the third in Norway (Eriksen, Bruusgaard & Knardahl, 2003).

Without doubt, WRMSs not only affect NAs’ health but also the quality of care they are able to provide to the elderly (Cheung et al., 2018). WRMSs have been reported as one of the leading reasons for NAs to quit their jobs (Cheung et al., 2018; Nelson & Baptiste, 2006). Castle & Engberg (2005) reported almost 90% of NAs working in NHs having left their jobs in one year. There is evidence that this turnover has led to a decline in the quality and continuity of care, an increase in the recruitment of less experienced NAs, more psychological distress for some elderly, and greater expenditure for facilities (Knapp & Missiakoulis, 1983). As a result, there is an urgent need to reduce WRMSs in NAs.

In spite of the high prevalence of shoulder WRMSs in NAs working in NHs, there has been little research to identify their associated factors. Less than half of the studies (45.5%, *n* = 60) reviewed by Davis & Kotowski (2015), examined the WRMSs in shoulders among the nursing personnel in hospitals, long term facilities or home healthcare. Out of these 60 studies, approximate 15% of them (*n* = 9) assessed the risk factors on the WRMSs in shoulders and only two studies including the NAs from hospitals (Davis & Kotowski, 2015). Unfortunately, there was no study focusing on the factors associated with shoulder WRMSs among the NAs working in NHs.

Factors associated with shoulder WRMSs among NAs in NHs might be unique because of their high prevalence of WRMSs, and possibly country specific. Comparing with the NAs working in hospitals and home care, NH NAs have a greater rate of WRMSs (Meyer & Muntaner, 1999; Jensen, 1987). This could be related to their demanding care-related activities (Ching et al., 2017) frequently involving shoulder muscles, such as heavy lifting, handling of loads with high force, or working in awkward postures in upper extremity (Linaker & Walker-Bone, 2015). Moreover, there are cultural differences in various aspects like common responses to musculoskeletal symptoms (Madan et al., 2008) and workplaces (Lee et al., 2012). Among the nine studies reviewed by Davis & Kotowski (2015) on shoulder WRMSs in hospital settings, only one study was conducted in Asian (i.e., Japan), and the rest in western countries. Both Asian (Smith et al., 2003a; Smith et al., 2003b) and western studies (Lamy et al., 2014; Alexopoulos, Burdorf & Kalokerinou, 2003; Pahlevan et al., 2014) have reported ergonomic factors such as hard physical load and psychosocial factors such as stress were the significant risk factors for shoulder WRMSs (Davis & Kotowski, 2015). Thus, participatory ergonomics programs have been proposed to prevent WRMSs (Magnavita, 2009a; Magnavita et al., 2007). In addition, the personal factors such as increasing age was an identified significant risk factor on shoulder WRMSs (Magnavita, 2009b; Shiue et al., 2008). Obesity (Luime et al., 2004), anxiety, depression, job strain (Magnavita, 2009b), and environmental discomfort (Magnavita et al., 2011) werer also found associated with shoulder WRMSs in western context. However, two Western studies (Warming et al., 2009; Moreira et al., 2014) have found no significant factors associated with shoulder WRMSs. Thus, the purpose of this study was to determine the prevalence of shoulder WRMSs and identify associated risk factors with shoulder WRMSs among NAs working in NHs in an Asian context. The study attempted to address the research question of “What were the associated factors of shoulder WRMSs among NAs working in NHs?” We hypothesized that personal, work and workstyle factors would be associated with shoulder WRMSs.

## Methods

### Study design and sampling

This was a cross-sectional survey study (Cheung et al., 2018). Data were collected from NAs working in Hong Kong NHs. NAs who were Chinese-speaking full-time employees and had worked for at least one year in the NHs were included. The exclusion criteria were those who were pregnant, working on night duty, suffering from serious medical pathological illnesses, such as cancer, or had received treatment for WRMSs in different body parts (including shoulders) that had ended less than one month before the study (Cheung et al., 2018). Convenience sampling was used to recruit NHs located in all three regions of Hong Kong: Hong Kong Island, Kowloon Peninsula, and the New Territories. 365 nursing homes approached and 440 NAs from 47 NHs (60–90% response rate from each NH) participated in this study in 2014–2015 (Cheung et al., 2018).

### Data collection

Ethical approval was obtained from The Human Subjects Ethics Sub-committee of the Hong Kong Polytechnic University (reference number: HSEARS20190722001). The trained research personnel explained the study and NAs signed the consent form. The NAs completed the questionnaire independently during their working hours. The research personnel assisted the NAs to complete the questionnaire if needed (Cheung et al., 2018).

### Measures

The questionnaire “Nursing Assistants Musculoskeletal Symptoms Questionnaire consists of eight parts to investigate the relationships between shoulder WRMSs, the dependent variable, and seven independent variables: demographic data, perceived physical exertion (PE), workstyle, ergonomic and manual handling knowledge, perceived ergonomic exposure, psychosocial factors, and other work-related factors (Cheung et al., 2018). A panel of four experts, two nurses, an occupational therapist, and a physiotherapist, was invited to evaluate the content validity of the questionnaire. A Content Validity Index (CVI) of 0.99 (Cheung et al., 2018) was obtained.

#### Dependent variable

##### Self-reported musculoskeletal symptoms

An adapted part of the Standardized Nordic Musculoskeletal Questionnaire (NMQ) was used to assess the prevalence of self-reported WRMSs, specifically pain, aches, or discomfort in shoulders. The participants were also asked if these symptoms were work-related. The responses were dichotomous (yes or no) (Cheung et al., 2018). The NMQ has been reported as valid and reliable for use with Hong Kong nursing personnel (Cheung et al., 2006).

#### Independent variables

##### Perceived Physical Exertion (PE)

Borg’s rating of PE scale (Borg, 1982), a 10-point scale (0 = “nothing at all” and 10 = “very, very strong”) was used to assess the intensity levels of the participants’ physical activity in the workplace by responding to a question “How would you rate your physical exertion in performing different activities during your working day?”. The sum of scale was used for analyzed. Borg (1982)’s rate of PE has been used commonly to examine physical activity intensity level in workplace studies (Roquelaure et al., 2014; Jakobsen et al., 2014).

##### Workstyle

A 24-item modified version of the Workstyle Short Form was used to assess each worker’s response to the increased work demands (Szeto et al., 2010; Cheng et al., 2014). This included five subscales: working through pain (6 items), social reactivity (5 items), workplace stressors (8 items), self-imposed workplace / workload (3 items), and breaks (2 items). Each item was scored on a five-point Likert scale (0 = almost never, 4 = almost always) (Feuerstein & Nicholas, 2006). The Workstyle Short Form was modified to assess all body parts. The sum of all items and each of the five subscales were analyzed. High frequencies of adverse workstyles were reflected by a high score. The modified Workstyle Short Form was found to be reliable with an overall Cronbach’s alpha of .92 (Cheung et al., 2018).

##### Ergonomic and manual handling knowledge

A set of 21 questions was used to measure the knowledge about ergonomic principles, manual handling, and how participants were able to apply this knowledge in practical situations (Ergonomics Technical Advisory Group, 2005; Hong Kong Labour Department, 2004; Nelson, Motacki & Menzel, 2009; Waters et al., 2009). The correct responses were summed up as a score for the knowledge to be analyzed (Cheung et al., 2018).

##### Perceived Ergonomic Exposures (EEs)

Two subscales, EE-contribution to WRMSs (measured by Yes or No responses) and EE-encounters frequency (measured by a four-point Likert scale from 0 = never to 4 = always), were used to measure the participants’ perceived EEs (Ergonomics Technical Advisory Group, 2005; Hong Kong Labour Department, 2004; Nelson, Motacki & Menzel, 2009; Szeto et al., 2010; Waters et al., 2009). Nine items were used to address the commonly identified EEs of awkward postures, static postures, repetitive movements, working tools that were poorly maintained, and forceful use of tools. The sum of each subscale was analyzed. High scores showed that the NAs’ WRMSs were related to highly frequent encounters of EEs. The Cronbach’s alpha was .82 for EE-contribution to WRMSs and .83 for EE-encounters frequency (Cheung et al., 2018).

##### Psychosocial factors

A job content questionnaire (JCQ) (Karasek et al., 1998), a four-point Likert scale, from strongly disagree to strongly agree (Karasek, 1997), was used to study seven subscales: decision authority (three items), skill discretion (six items), psychological job demand (nine items), physical job demand (five items), supervisor support (four items), co-worker support (five items), and residents / their family support (four items). JCQ was found to have high validity in another study (Karasek, 1997). The Cronbach alpha of JCQ was .75 (Cheung et al., 2018) which showed that JCQ is reliable. The sum of subscales was used for data analyses.

##### Other work-related factors

Work-related factors were assessed by three individual single items, job satisfaction, job stress, and intention to leave the job. Job satisfaction and job stress were indicated by a four-point Likert scale, and intention to leave the job was indicated with dichotomous responses. Also, the participants’ current work ability was self-assessed by rating from 0 (completely no work ability) to 10 (completely full ability). Apart from this, three options (no, not sure, and surely yes) were used to assess the participants’ perceptions of whether their health conditions would allow them to work in the coming two years (Cheung et al., 2018).

#### Demographic data

Age, gender, marital status, education level, self-rated health, exercise patterns, smoking habits, history of surgery, job title, overtime work, lifting/ transferring training, years of work experience, body mass index (kg/m^2^), and waist-to-hip ratio were included as demographic data in the study (Cheung et al., 2018). 

### Data analysis

IBM SPSS Statistics version 26 (IBM Corp., Armonk, NY, USA) was used for data analysis. There was .22%–1.36% of missing data for the independent variable while there were no missing data for shoulder WRMSs (Cheung et al., 2018). To compare NAs with and without shoulder WRMSs, chi-square and independent sample t-tests were used for the bivariate analysis. Significant associated factors were then analyzed further by multivariable logistic regression. Nonetheless, after multicollinearity screening, the total workstyle variable had high correlation .91 with working through pain subscales. Working through pain was selected for logistic regression analyses instead of the total workstyle since other subscales in workstyle were not statistically significant. *P*-values < .05 were considered statistically significant in all analyses.

## Results

### Characteristics of the NAs

The participants’ personal and work-related characteristics have been presented elsewhere (Cheung et al., 2018). They were mostly female (98.0%, *n* = 431), married (79.8%, *n* = 351), with a high school education level (63.6%, *n* = 276), and had a mean age of 51.1 years (SD = 9.6). They rated their health status as “not good” (44.8%, *n* = 197) or “fair” (39.5%, *n* = 174), and tended to be overweight (mean body mass index (BMI) = 24.7, SD = 3.4). Approximately 90% of the participants worked with the job title “personal care worker” (90.9%, *n* = 400), with an average of 10.4 years of working experience (SD = 6.4). Most of them had received lifting and transferring training (90.4%, *n* = 397), and 74.5% (*n* = 327) of them did not need to work overtime. 70% (*n* = 28) health workers and 51.3% (*n* = 205) personal care workers reported that they were experiencing shoulder WRMSs.

### Factors associated with shoulder WRMSs by Bivariate analysis

53% (*n* = 233) of the NAs reported experiencing WRMSs in their shoulders. Table 1 indicates the factors significantly associated with shoulder WRMSs. When personal characteristics were considered, it was surprising that NAs with shoulder WRMSs (*M* = 24.3) had significantly lower BMIs than those without (*M* = 25.8). NAs with shoulder WRMSs were significantly more likely to rate their health status as ‘bad’. On the other hand, age, gender, marital status, educational level, exercise habits, smoking habits, history of surgery and waist-hip ratio were not associated with shoulder WRMSs.

**Table 1:** Factors significantly associated with WRMSs on shoulders among NAs by bivariate analysis (*N* = 440).

	With WRMSs on shoulders	Without WRMSs on shoulders		
Personal characteristics	Number (%)	Number (%)	Mean (SD)	*p*-value
Self-rated Health Status	*n* = 233	*n* = 207	–	.005^**^
Bad	126 (54.1%)	81 (39.1%)		
Fair	83 (35.6%)	91 (44.0%)	–	
Good	24 (10.3%)	35 (16.9%)	–	
BMI	*n* = 233	*n* = 277	24.3 (3.08)	.020^*^
			25.1 (3.77)	
Work characteristics				
Job Title	*n* = 233	*n* = 207	–	
Personal care workers (PCW)	205 (88.0%)	195 (94.2%)	–	.023^*^
Health workers (HW)	28 (12.0%)	12 (5.8%)	–	
Ergonomic Exposures (EEs) -	*n* = 232	*n* = 193	16.3 (7.36)	<.001^***^
Encounters frequency			12.8 (8.41)	
Ergonomic Exposures (EEs) -	*n* = 231	*n* = 193	5.07 (2.51)	<.001^***^
Contribution to WRMSs			3.98 (2.74)	
Self-rated job stress	*n* = 231	*n* = 207	–	.005^**^
Stressful/ very stressful	174 (75.3%)	130 (62.8%)		
No stress/ not very stressful	57 (24.7%)	77 (37.2%)		
Intention to leave	n=231	*n* = 206	–	.003^**^
Yes	96 (41.6%)	58 (28.2%)		
No	135 (58.4%)	148 (71.8%)		
Workstyle characteristics				
Workstyle (total)	*n* = 232	*n* = 207	28.4 (14.7)	0.01^*^
			14.5 (16.1)	
Working Through Pain	*n* = 232	*n* = 207	10.4 (4.79)	<.001^***^
			7.77 (5.37)	

**Notes.**

* *p* < .05.

** *p* < 0.01.

*** *p* < .001.

The work characteristics were compared for NAs with and without shoulder WRMSs. Health workers were significantly more likely to have WRMSs than personal care workers. They also regarded their work as more stressful, had higher intention to quit their jobs, and higher scores for both EE-contribution to WRMSs and EE-encounters frequency subscales, as well as total workstyle scores and the working through pain subscale. On the contrary, lifting training, working overtime, perceived PE , job satisfaction and expecting their health conditions to affect their working ability in the following two years were not related to the shoulder WRMSs. Additionally, there were no differences between NAs with or without shoulder WRMSs for knowledge of ergonomic and manual handling, years of work experience, perceived current working ability, all workstyle subscales except working through pain and all JCQ subscales of psychosocial factors.

### Multivariable logistic regression

Table 2 shows the results of the multivariable logistic regression analyses. Due to multi-collinearity, working through pain was selected instead of workstyle (total) for the final regression model. With the adjustment of age and gender, with an increase in one unit of BMI (kg/m^2^), the NAs had .931 times less likely to have shoulder WRMSs. NAs with the job title “health workers”, were 2.72 times more likely to have shoulder WRMSs than NAs with the job title “personal care worker”; and NAs with working-with-pain workstyle behaviors had 1.06 times more likely to develop WRMSs in their shoulders than those who did not demonstrate such behaviors.

**Table 2:** Logistic regression analysis to identify predictors for WRMSs on shoulders in NAs working in nursing homes, adjusted for age and gender (*N* = 440).

Adjusted for age and gender, with nagelkerkes’ *R*^2^ = 12.3%			
Variables	Odds ratio	95% CI	*p*-value
Gender			
Female	2.62	.422–16.3	.301
Male (reference)	1	–	–
Age	.999	.977–1.02	.960
BMI	.931	.874–.991	.025^*^
Self-rated Health Status			
Bad	1.54	.804–2.97	.192
Fair	1.09	.562–2.09	.809
Good (reference)	1	–	–
Job Title			
Health workers	2.72	1.18–6.25	.018^*^
Personal care workers (reference)	1	–	–
EE–contribution to WRMSs	.995	.880–1.13	.938
EE–encounter frequency	1.04	.998–1.08	.062
Workstyle-Work through pain	1.06	1.01–1.11	.020^*^
Self-rated Job Stress			
Stressful/ very stressful	1.04	.643–1.67	.882
Not stress/ not very stressful (reference)	1	–	–
Intention to leave			
Yes	1.15	.720–1.84	.558
No (reference)	1	–	–

**Notes.**

* *p* < .05.

## Discussion

This is probably the first study to have investigated predictors for shoulder WRMSs with NAs in an Asian nursing home context. Our study found that BMI, being in the position of health worker, and adopting an adverse workstyle of working through pain were the predictors.

BMI was found to be associated inversely with shoulder WRMSs for the NAs in our study. For Asian people (Center For Health Protection, 2019), BMIs between 23.0–24.9 kg/m^2^ and 25.0 kg/m^2^ or above are classified as overweight and obese, respectively (Center For Health Protection, 2019). A further examination of our data showed that NAs with shoulder WRMSs had lower BMI (*M* = 24.3 kg/m^2^) (i.e., overweight) than those without (*M* = 25.1 kg/m^2^) (i.e., obese). Our results were partially in agreement with those of Viester and colleagues (2013), who found that being overweight was positively associated with overall musculoskeletal symptoms in the general working population. However, the inverse association between BMI and shoulder WRMSs was an unexpected result. To our knowledge, only Tsuritani and colleagues (2002) have found a similar result. They found that a higher BMI was associated with less shoulder pain in Japanese middle-aged women. This similar finding might be because the participants involved were Asian women, with similar mean BMIs (such as *M* = 23.8 kg/m^2^ for Chinese, *M* = 22.7 kg/m^2^ for Japanese Global Health Observatory, 2016). In addition, our study involved mostly women, as did the Tsuritani and colleagues study (2002). This unexpected result might have been because stronger muscle mass and strength are associated with increasing BMI (Pasdar et al., 2019) and even obesity (Lafortuna et al., 2005). It has been found that stronger shoulders may lead to less shoulder problems in heavier women, with weight positively associated with shoulder strength (Hughes et al., 1999). However, our Asian findings were contradictory to those in western contexts (Bodin et al., 2012; Monteiro et al., 2015; Nilsen, Holtermann & Mork, 2011; Luime et al., 2004; Pelissier et al., 2014; Viester et al., 2013). Their findings were explained by the effect of metabolic factors, with obesity associated with the development of osteoarthritis, rheumatic diseases (Viester et al., 2013) and chronic low-grade systemic inflammation (Nilsen, Holtermann & Mork, 2011). Nevertheless, the relationship between BMI and WRMSs on shoulders is not conclusive. Some studies even found no significant relationship between them (Weiner et al., 2015; Wami, Dessie & Chercos, 2019; Tantawy, 2019). The inconsistent results could be related to heterogeneity of the working groups under study. For instance, the positive relationships between the risks of shoulder WRMSs and BMI were found in large working populations (Bodin et al., 2012; Viester et al., 2013), while those showing no such significant association were conducted with housekeepers (Wami, Dessie & Chercos, 2019) and nurses (Weiner et al., 2015). Our study is the first of its kind to have investigated NAs in NHs. The inconsistent findings could also be as a result of differences in average BMIs in various countries. According to WHO, the mean BMIs in France (M = 25 kg/m^2^) and Germany (*M* = 26.6 kg/m^2^) are higher than that in China (*M* = 23.84 kg/m^2)^ andJapan (*M* = 22.74 kg/m^2^) [58]. Furthermore, the studies reporting the risk of shoulder problems as increasing with BMI (Bodin et al., 2012; Nilsen, Holtermann & Mork, 2011; Luime et al., 2004; Pelissier et al., 2014; Viester et al., 2013) or showing no significant results (Weiner et al., 2015; Wami, Dessie & Chercos, 2019; Tantawy, 2019) were all conducted in non-Asian countries. Further studies should evaluate the relationship between shoulder strength and WRMSs in shoulders.

Our study also found that job title was another significant predictor for shoulder WRMSs. This could be explained by the positive association of workload and shoulder WRMSs (Lagerstrom et al., 1995; Arvidsson et al., 2006; Feng et al., 2014) especially in women (Hanvold et al., 2014). In Hong Kong, NAs are classified as either health workers (HWs) or personal care workers (PCWs). While both of these are health-care positions, HWs have higher educational qualifications and advanced training (Social Welfare Department, 2005; Hong Kong Health Care Federation, 2020). The reason for HWs being more prone to shoulder WRMSs might be explained by the difference in their job responsibilities. Since HWs have higher qualifications, they are considered to be more competent than PCWs and are required to provide advanced nursing care to the residents, which may result in heavier workloads. Both HWs and PCWs are responsible for rendering basic care to residents, but HWs have to provide additional advanced care like wound dressing, nasogastric tube feeding, and dispensing medicines (Social Welfare Department, 2013). With more and a greater complexity of tasks, they are more likely to increase the use of their shoulder muscles, hence the risk of developing WRMSs in shoulders. Apart from activities that involve handling residents, HWs are also responsible for recording their health history and information (Social Welfare Department, 2013). To our knowledge, most of the NHs in Hong Kong are still using paper-based documentation. This kind of desk-based task might contribute to WRMSs in shoulders. Leonard and colleagues (2010) found that prolonged writing tasks for 30 min would increase upper trapezius muscle activity, which can lead to increased muscle tension and cause reflex guarding of the muscle (Greig, Straker & Briggs, 2005). Based on this mechanism, discomfort or pain in the shoulders might be attributed to an increase of trapezius muscle activity from prolonged paperwork. Thus, HWs might be exposed to higher workloads and more risky tasks than PCWs, which could increase their vulnerability to WRMSs. Nevertheless, it is difficult to compare our findings with others since most of the previous studies did not classify NAs by different job titles.

Last but not least, working through pain was also a significant predictor for NAs experiencing shoulder WRMSs. Working through pain is an adverse workstyle behavior in which an individual keeps working despite increasing pain and other WRMSs (Maakip, Keegel & Oakman, 2015). Our finding was consistent with other studies, that working through pain was a predictor for at least one body part having WRMSs for NAs in NHs (Cheung et al., 2018); and upper extremity WRMSs in the general population (Meijer, Sluiter & Frings-Dresen, 2008). In addition, working through pain contributed the largest mediating effect between work exposure and upper extremity pain, including shoulders (Van den Heuvel et al., 2007). Feuerstein, Huang & Pransky (1999) explained the linkage of working through pain and WRMSs, suggesting that it is a barrier to the recovery of muscle tissue. One study found that even when NAs were entitled to sick leave, they would continue to work because they did not want their co-workers to have to take up their work (Ching et al., 2017). As well, an intervention study of low-skilled workers (Cheung et al., 2019) suggested that improving the workstyle of working through pain could significantly reduce the number of body parts with WRMSs (Cheung et al., 2019). Redesigning the work may be the essential element to maintaining the musculoskeletal health of NAs. In order to construct a direct and effective intervention, changes in workstyles of both the NHs and NAs should be taken into consideration. It would be worthwhile for employers to establish healthy workstyles in NHs, such as providing more breaks, and reducing the work demands. Furthermore, employers could consider organizing multidisciplinary exercise programs aiming to teach NAs how to prevent and manage their WRMSs through stretching exercises and accurate workstyle knowledge. An eight-week community-based multidisciplinary exercise program for low-skilled workers has demonstrated the reduction of WRMSs symptoms, incidences of working through pain and improved self-rated health status (Cheung et al., 2019). Similar programs could be adopted in NHs to enhance their NAs’ musculoskeletal health.

Few limitations were found in this study. Its cross-sectional nature excludes the cause-and-effect relationships among the risk factors and shoulder WRMSs. The exclusion of NAs receiving active treatment for musculoskeletal problems might have led to an underestimation of the prevalence of the WRMSs in shoulders. Furthermore, we found a medium power of 0.6 to detect the statistical significance of shoulder WRMSs on the job title. 60 health workers are required to reach the power of 0.80 and *p* < 0.05.

## Conclusion

Our findings indicated 53% NAs in NHs reported shoulder WRMSs. This study has identified BMI, job title and working through pain as the predictors for shoulder WRMSs in NAs working in NHs. Further studies should examine the relationships among shoulder strength, BMI, and shoulder WRMSs. In addition, it might not be appropriate to assume that one kind of prevention program will fit all workers, because NAs with different job titles might experience unique job demands and risk factors. The workstyle of working through pain has been well identified as a risk factor for WRMSs in different body parts. Redesigning jobs with more breaks and reducing the work demand and introducing multidisciplinary exercise programs should be explored further to improve workstyle behaviors and enhance the musculoskeletal health of NAs.

##  Supplemental Information

10.7717/peerj.11152/supp-1 Supplemental Information 1 Raw Data.

10.7717/peerj.11152/supp-2 Supplemental Information 2 Study Questionnaire (English).

10.7717/peerj.11152/supp-3 Supplemental Information 3 Study Questionnaire (Chinese).

## Additional Information and Declarations

### Data Availability

The following information was supplied regarding data availability: Raw measurements are available in the Supplemental Files.
