# Supplementary material for: Predictors of work-related musculoskeletal symptoms in shoulders among nursing assistants working in nursing homes
Source: PeerJ. 2021 May 3;9:e11152. doi: 10.7717/peerj.11152 (PMC8101459; doi:10.7717/peerj.11152)
Supplement: Supplemental Information 2 [file peerj-09-11152-s002.doc]

# **Section I** **Your Personal Information**

# ***(Please fill in your answers to the questions or put a √ in the appropriate box)***

1. Are you male or female? _0_🞎 Male

_1_🞎 Female

1. How old are you? ___________ years old
2. What is your height? __________ cm (or ________ feet)
3. What is your weight? __________ kg (or ________ lbs)
4. What is your marital status?

_0_🞎 Single _1_🞎 Married _2_🞎 Divorced/Separated _3_🞎 Widowed _4_🞎 Other (SPECIFY: ________________)

1. What is your educational level?

_0_🞎 Primary _1_🞎 Secondary _2_🞎Post-secondary _3_🞎 Bachelor or above

1. Have you had any serious illnesses before? _0_🞎 No _1_🞎 Yes ☞ please specify: _____________
2. Do you currently have any serious illnesses? _0_🞎 No _1_🞎 Yes ☞ please specify: _____________
3. Have you ever had any surgery? _0_🞎 No _1_🞎 Yes ☞ what kind of surgery? ______________
4. Are you pregnant right now? _0_🞎 No _1_🞎 Yes _2_🞎 Not applicable
5. In general, would you say your health is:

_0_🞎 Poor _1_🞎 Fair _2_🞎Good _3_🞎 Very good _4_🞎 Excellent

1. Do you exercise at a moderate intensity (which makes you slightly sweaty) for 20 minutes 3 times a week or more?

_0_🞎 Never _1_🞎 Occasionally _2_🞎 Often _3_🞎 Always

1. What is your smoking history:

_0_🞎 Never smoked _1_🞎 Currently smoking _2_🞎 Former smoker ☞ Year you quit: ________

1. What is your drinking alcohol history:

_0_🞎 Never drank _1_🞎 Currently drinking _2_🞎 Former drinker ☞ Year you quit: ________

1. Do you perform stretching exercise daily? _0_🞎 No _1_🞎 Yes
2. Do you perform muscle strengthening exercise daily? _0_🞎 No _1_🞎 Yes
3. Have you received lifting and transferring residents training? _0_🞎 No _1_🞎 Yes
4. Your current position is a _0_🞎 Personal health worker _1_🞎 Health worker
5. How long have you been working on this current position? ____________ years
6. On average, did you work overtime? _0_🞎 No _1_🞎 Yes

# **Section II Your Musculoskeletal Problems**

1. Identify the musculoskeletal problems in Figures 1 and 2.

# Instructions:

# Circle the name of the area(s) that you have problem(s) now (such as aches, pains, discomfort, or numbness)

1. Put a X in the body where you have problem(s)

**
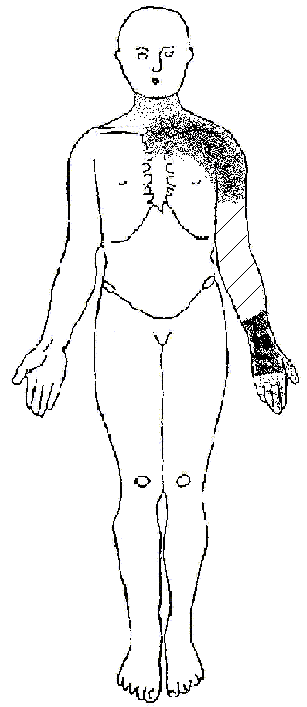

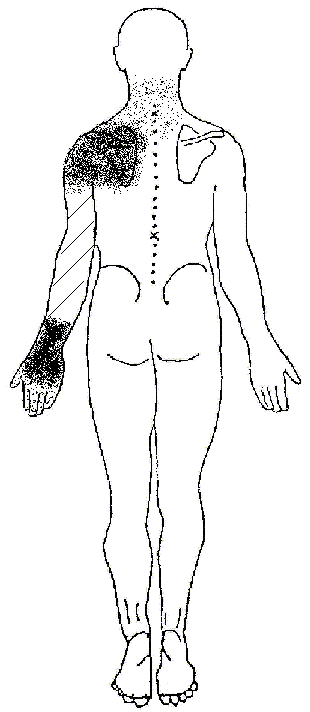
**

Shoulders

Elbows / Forearms

Wrists / Hands

Neck

Fingers

**Figure 1**


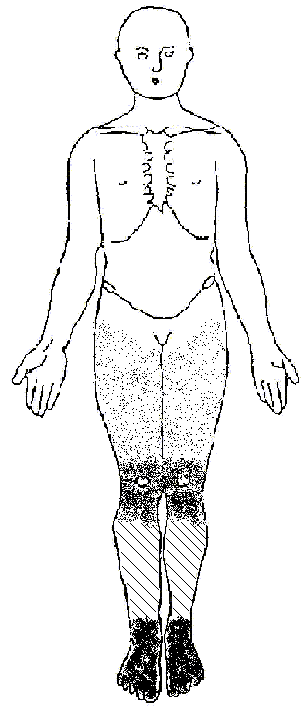

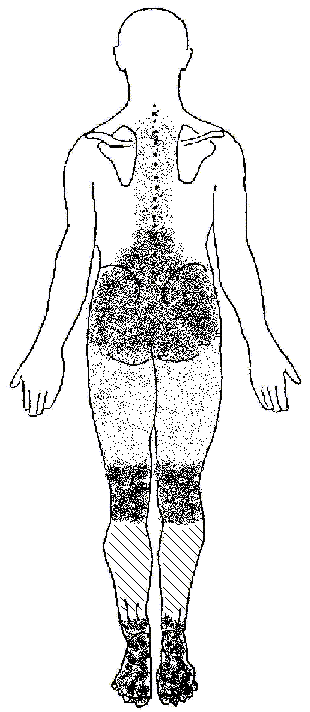


Upper Back

Lower Back

Hips / Thighs

Knees

Calves

Ankles / Feet

**Figure 2**

1. Please put a **√** in the appropriate box for shoulder problems

|  | **Shoulder(s)** |
| --- | --- |
| How long have you had the problems? | _0_🞎 <1 month  _1_🞎 1- 6 months  _2_🞎 7-11 months  _3_🞎 ≧1 – 3 ＜years  _4_🞎 ≧3 – 5＜years  _5_🞎 ≧5 years |
| Are the problems caused by your work? | _0_🞎 YES  _1_🞎 NO |

# **Section III Your Perceived Physical Exertion (PE)**

1. Please estimate the frequency and perceived physical exertion on the main work activities that you do daily.

* Please use the following scale to circle the perceived physical exertion

| 0 1 2 3 4 5 6 7 8 9 10  Nothing Very Fairly Moderate Somewhat Hard Very Very  at all Light Light Hard Hard Very Hard |
| --- |

|  | Types of Main Work Activity | Perceived Physical Exertion* |
| --- | --- | --- |
| 1. | Transferring patients between bed/wheelchair | 0 1 2 3 4 5 6 7 8 9 10 |
| 2. | Transferring patients between wheelchair/toilet | 0 1 2 3 4 5 6 7 8 9 10 |
| 3. | Transferring patients between bath cart/bed | 0 1 2 3 4 5 6 7 8 9 10 |
| 4. | Repositioning patients in wheelchair/chair/bed | 0 1 2 3 4 5 6 7 8 9 10 |
| 5. | Dressing or undressing patients | 0 1 2 3 4 5 6 7 8 9 10 |
| 6. | Changing incontinent diaper | 0 1 2 3 4 5 6 7 8 9 10 |
| 7. | Restraining patient | 0 1 2 3 4 5 6 7 8 9 10 |
| 8. | Bathing patients | 0 1 2 3 4 5 6 7 8 9 10 |
| 9. | Lifting and carrying meal trays | 0 1 2 3 4 5 6 7 8 9 10 |
| 10. | Feeding bedridden patient | 0 1 2 3 4 5 6 7 8 9 10 |
| 11. | Making bed with patient | 0 1 2 3 4 5 6 7 8 9 10 |
| 12. | Making bed without patient | 0 1 2 3 4 5 6 7 8 9 10 |
| 13. | Trying to prevent the patient from falling | 0 1 2 3 4 5 6 7 8 9 10 |
| 14. | Lifting fallen patient from floor using lifting device | 0 1 2 3 4 5 6 7 8 9 10 |
| 15. | Moving patients who are completely unable to move by themselves | 0 1 2 3 4 5 6 7 8 9 10 |
| 16. | Moving cooperated patient | 0 1 2 3 4 5 6 7 8 9 10 |
| 17. | Uncoordinated when working with colleagues to move residents | 0 1 2 3 4 5 6 7 8 9 10 |
| 18. | Transport laundry to washing machine/dryer | 0 1 2 3 4 5 6 7 8 9 10 |
| 19. | Hang patients’ clothes for drying | 0 1 2 3 4 5 6 7 8 9 10 |
| 20. | Fold patients’ clothes after drying | 0 1 2 3 4 5 6 7 8 9 10 |
| 21. | Cleaning work environment (e.g. fans, patients’ tables, walls) | 0 1 2 3 4 5 6 7 8 9 10 |
| 22. | Pushing damaged or poorly maintained tools | 0 1 2 3 4 5 6 7 8 9 10 |
| 23. | Others, please specify: ____________ | 0 1 2 3 4 5 6 7 8 9 10 |

# **Section IV Workstyle**

1. Please complete the following items by checking the boxes that describe your experience at work.

|  |  | Almost never | Rarely | Sometimes | Frequently | Almost always |
| --- | --- | --- | --- | --- | --- | --- |
| 1. | I keep working when I feel aching or discomfort, so that the quality of my work will not be affected. | _0_⬜ | _1_⬜ | _2_⬜ | _3_⬜ | _4_⬜ |
| 2. | My neck/shoulders/hands/arms/back/hips/thighs/calves/feet get tired at work. | _0_⬜ | _1_⬜ | _2_⬜ | _3_⬜ | _4_⬜ |
| 3. | My neck/shoulders/hands/arms/back/hips/thighs/calves/feet get tired at work. | _0_⬜ | _1_⬜ | _2_⬜ | _3_⬜ | _4_⬜ |
| 4. | I feel aching while at work | _0_⬜ | _1_⬜ | _2_⬜ | _3_⬜ | _4_⬜ |
| 5. | As I do not know how to relieve my aching neck/shoulders/hands/arms/back/hips/ thighs/calves/feet, I keep on working with pain. | _0_⬜ | _1_⬜ | _2_⬜ | _3_⬜ | _4_⬜ |
| 6. | I do not know how to eliminate or relieve various symptoms of my neck/shoulders/hands/arms/back/hips/thighs/calves/feet。 | _0_⬜ | _1_⬜ | _2_⬜ | _3_⬜ | _4_⬜ |
| 7. | My neck/upper extremities/back/lower extremities (over or more places) may make some abrupt, fierce, fast or sudden movements. | _0_⬜ | _1_⬜ | _2_⬜ | _3_⬜ | _4_⬜ |
| 8. | I cannot interrupt my work because my other team members will be unhappy with me. | _0_⬜ | _1_⬜ | _2_⬜ | _3_⬜ | _4_⬜ |
| 9. | I cannot interrupt my work, because it would disappoint my colleagues or increase their workload. | _0_⬜ | _1_⬜ | _2_⬜ | _3_⬜ | _4_⬜ |
| 10. | I cannot interrupt my work, because it would affect my appraisal, promotion, and/or cause me to lose my job. | _0_⬜ | _1_⬜ | _2_⬜ | _3_⬜ | _4_⬜ |
| 11. | If I take a break from work for relaxation or physical exercises, my colleagues/boss will be unhappy with me. | _0_⬜ | _1_⬜ | _2_⬜ | _3_⬜ | _4_⬜ |
| 12. | Although I have worked very hard, I still do not know whether my work has been duly recognized. | _0_⬜ | _1_⬜ | _2_⬜ | _3_⬜ | _4_⬜ |
| 13. | If I have not finished my work, my boss will give me a hard time. | _0_⬜ | _1_⬜ | _2_⬜ | _3_⬜ | _4_⬜ |
| 14. | If I communicate some problems to the supervisor – for instance, that some coworkers have not been working hard enough – this has no effect, so I might as well just work harder. | _0_⬜ | _1_⬜ | _2_⬜ | _3_⬜ | _4_⬜ |
| 15. | I feel very depressed, as my boss’ expectations on the quality of work are different from mine. | _0_⬜ | _1_⬜ | _2_⬜ | _3_⬜ | _4_⬜ |
| 16. | I have too many deadlines and I can never finish my work. | _0_⬜ | _1_⬜ | _2_⬜ | _3_⬜ | _4_⬜ |
| 17. | Although I arrange my work in good order so that I can finish it before the deadline, things change so frequently that I have to work even harder to finish it on time. | _0_⬜ | _1_⬜ | _2_⬜ | _3_⬜ | _4_⬜ |
| 18. | My work schedule is hard to control. | _0_⬜ | _1_⬜ | _2_⬜ | _3_⬜ | _4_⬜ |
| 19. | I feel pressure at work. | _0_⬜ | _1_⬜ | _2_⬜ | _3_⬜ | _4_⬜ |
| 20. | I motivate myself to work harder and set up targets that are higher than those my boss and other colleagues have expected. | _0_⬜ | _1_⬜ | _2_⬜ | _3_⬜ | _4_⬜ |
| 21. | If my colleagues fail to do their work, I have to assume more responsibilities. | _0_⬜ | _1_⬜ | _2_⬜ | _3_⬜ | _4_⬜ |
| 22. | Others tell me to slow down and not to work so intensely. | _0_⬜ | _1_⬜ | _2_⬜ | _3_⬜ | _4_⬜ |
| 23. | During my regular workday I take breaks to do some stretches. | _0_⬜ | _1_⬜ | _2_⬜ | _3_⬜ | _4_⬜ |
| 24. | While at work I occasionally stop working to take a break. | _0_⬜ | _1_⬜ | _2_⬜ | _3_⬜ | _4_⬜ |

# **Section V Ergonomic and Manual Handling Knowledge**

25. Please circle the right statement.

| 1. | When moving dependent residents, if your colleagues cannot help you move the residents, it is acceptable and appropriate for you to move the residents by yourself | True | False |
| --- | --- | --- | --- |
|  | Bending your body to take care of patients is not one of the risk factors for musculoskeletal problems | True | False |
|  | When lifting patients or other objects, you should bend your knees (not waist) and use your feet (not your back) to lift | True | False |
|  | When moving residents or other objects, you should stand as far as possible from the person/object you will be lifting | True | False |
|  | You should try to assist the transfer of residents on the stronger side of the residents | True | False |
|  | It’s a good habit to find time to take a break or stretch muscle | True | False |
|  | **It’s a good habit to find time to stop or stretch your bones** | True | False |
|  | Excessive force and repetitive movements are one of the risk factors for musculoskeletal problems | True | False |
|  | Leaning forward for a long time is an inappropriate posture which is a risk factor for musculoskeletal problems | True | False |
|  | Excessive turning of the body is bad posture | True | False |
|  | Sudden pulling of draw sheet while transferring residents will increase the risk of musculoskeletal problems | True | False |
|  | Pushing damaged or poorly maintained transferring devices (such as wheelchairs, bath beds) can cause musculoskeletal problems | True | False |
|  | Using trolleys, wheelchairs, shower/commode chairs, or mechanical lifting devices can eliminate or reduce the effort of lifting. | True | False |
|  | When changing bed sheets, the ideal working height is at your waist level. | True | False |
|  | By adjusting the height of the bed to the height of the wheelchair, you can move the residents steadily laterally and reduce the lifting of the residents. | True | False |
|  | You do not need to explain clearly to the residents before moving/moving because the residents will not understand. | True | False |
|  | Lifting between your hips and chest is the most powerful. | True | False |
|  | Pushing in/out is more powerful than moving left/right (across the body). | True | False |
|  | Sitting on the sofa/lying on the bed after returning home from work is the best way to relieve muscle fatigue. | True | False |
|  | Stretching must be done from head to toe in one go. | True | False |
|  | Stretching exercises must be done before and after work and during work to reduce musculoskeletal problems | True | False |

# **Section VI Perceived Ergonomic Exposures (EEs)**

26. Which one of the following descriptions do you think is the main cause of your musculoskeletal problems? Please indicate how frequently you have encountered these causes.

|  | Yes / No |  | Never Sometimes Often Always |
| --- | --- | --- | --- |
| 1. Sustaining static or awkward upper limb or upper body posture during work | _0_🞎 No _1_🞎 Yes |  | 0 1 2 3 |
| 2. Performing repetitive upper limb or upper body movements | _0_🞎 No _1_🞎 Yes |  | 0 1 2 3 |
| 3. Sustaining static or awkward lower limb or lower body posture during work | _0_🞎 No _1_🞎 Yes |  | 0 1 2 3 |
| 4. Performing repetitive lower limb or lower body movements | _0_🞎 No _1_🞎 Yes |  | 0 1 2 3 |
| 5. Exerting force in using certain instruments (e.g. forceful gripping of drawsheet to help repositioning) | _0_🞎 No _1_🞎 Yes |  | 0 1 2 3 |
| 6. Inappropriate arrangement of furniture such as beds, wheelchairs, tables (e.g. incorrect height of bed, bed placed against the wall) | _0_🞎 No _1_🞎 Yes |  | 0 1 2 3 |
| 7. Use damaged or poorly maintained tools (e.g. bath beds, wheelchairs, lift machines, etc.) | _0_🞎 No _1_🞎 Yes |  | 0 1 2 3 |
| 8. Physical factors in the working area (e.g. lighting, temperature, climbing stairs etc.) | _0_🞎 No _1_🞎 Yes |  | 0 1 2 3 |
| 9. Work stress or unsatisfied with current work | _0_🞎 No _1_🞎 Yes |  | 0 1 2 3 |

# **Section VII Psychosocial Factors**

28. The following questions are about **your job at work**. For some of these questions, you may find it difficult to choose the “correct” answer. Please ***CIRCLE*** the number which **best** describes the extent to which you agree or disagree with the following statements:

|  | **Strongly disagree** | **Disagree** | **Agree** | **Strongly agree** |
| --- | --- | --- | --- | --- |
| 1. My job requires that I learn new things. | 1 | 2 | 3 | 4 |
| 2. My job involves a lot of repetitive work. | 1 | 2 | 3 | 4 |
| 3. My job requires me to be creative. | 1 | 2 | 3 | 4 |
| 4. My job requires a high level of skill. | 1 | 2 | 3 | 4 |
| 5. I get to do a variety of different things on my job. | 1 | 2 | 3 | 4 |
| 6. I have an opportunity to develop my own special abilities | 1 | 2 | 3 | 4 |
| 7. My job allows me to make a lot of decisions on my own. | 1 | 2 | 3 | 4 |
| 8. On my job, I have little freedom to decide how I do my work. | 1 | 2 | 3 | 4 |
| 9. I have a lot of say about what happens on my job | 1 | 2 | 3 | 4 |
| 10. My job requires working very fast. | 1 | 2 | 3 | 4 |
| 11. My job requires working very hard. | 1 | 2 | 3 | 4 |
| 12. I am not asked to do an excessive amount of work. | 1 | 2 | 3 | 4 |
| 13. I have enough time to get the job done. | 1 | 2 | 3 | 4 |
| 14. I am free from conflicting demands that others make. | 1 | 2 | 3 | 4 |
| 15. My job requires long periods of intense concentration on the task. | 1 | 2 | 3 | 4 |
| 16. My tasks are often interrupted before they can be completed, requiring attention at a later time. | 1 | 2 | 3 | 4 |
| 17. My job is very hectic. | 1 | 2 | 3 | 4 |
| 18. Waiting on work from other people or departments often slows me down on my job. | 1 | 2 | 3 | 4 |
| 19. My supervisor is concerned about the welfare of those under her/him. | 1 | 2 | 3 | 4 |
| 20. My supervisor pays attention to what I am saying. | 1 | 2 | 3 | 4 |
| 22. My supervisor is helpful in getting the job done. | 1 | 2 | 3 | 4 |
| 23. My supervisor is successful in getting people to work together. | 1 | 2 | 3 | 4 |
| 24. People I work with are competent in doing their jobs | 1 | 2 | 3 | 4 |
| 25. People I work with take an interest in me. | 1 | 2 | 3 | 4 |
| 27. People I work with are friendly. | 1 | 2 | 3 | 4 |
| 28. The people I work with encourage each other to work together. | 1 | 2 | 3 | 4 |
| 29. People I work with are helpful in getting the job done. | 1 | 2 | 3 | 4 |
| 30. Patients and family members I work with take an interest in me. | 1 | 2 | 3 | 4 |
| 32. Patients and family members I work with are friendly. | 1 | 2 | 3 | 4 |
| 33. Patients and family members I work with encourage each other to work together. | 1 | 2 | 3 | 4 |
| 34. Patients and family members I work with are helpful in getting the job done. | 1 | 2 | 3 | 4 |
| 35. My job requires me to walk very fast on the road. | 1 | 2 | 3 | 4 |
| 36. My job requires lots of physical effort. | 1 | 2 | 3 | 4 |
| 37. I am often required to move or lift very heavy loads on my job | 1 | 2 | 3 | 4 |
| 38. My work requires rapid and continuous physical activity | 1 | 2 | 3 | 4 |
| 39. I am often required to work for long periods with upper body or upper extremity in physically awkward positions. | 1 | 2 | 3 | 4 |
| 40. I am required to work for long periods with lower body or lower extremity in physically awkward positions. | 1 | 2 | 3 | 4 |

# **Section VIII Other Work-related Factors**

**29. Please put a √ in the appropriate box that describe your views on current job.**

| 1. | I am satisfied with my current job | □ Very Satisfactory | □ Satisfactory | □ Unsatisfactory | □ Very Unsatisfactory |
| --- | --- | --- | --- | --- | --- |
| 2. | I feel stressful on my current job | □ Very Stressful | □ Stressful | □ Not Stressful | □ Not at all Stressful |
| 3. | I thought about quitting the job | □ Yes, I have thought of it | | □ No, I have not thought of it | |

| 4. Do you think you can continue to work for two years in your current position based on your health?  _1_ Impossible _2_ Not sure _3_ Sure |
| --- |

## THANK YOU FOR TAKING THE TIME

## TO COMPLETE THIS QUESTIONNAIRE
