# Supplementary material for: Predictors of work-related musculoskeletal symptoms in shoulders among nursing assistants working in nursing homes
Source: PeerJ. 2021 May 3;9:e11152. doi: 10.7717/peerj.11152 (PMC8101459; doi:10.7717/peerj.11152)
Supplement: Supplemental Information 3 [file peerj-09-11152-s003.doc]

# **第一部** **個人資料**

#

# ***(請填上你的答案或在適當方格中打上剔號「***✓***」)*** 對於某些問題，您可能難以選擇答案。很多的問題是沒有絕對的對或錯的答案，您只須選擇**最能形容**您的情況作為答案。

1. 性別: _0_🞎 男 _1_🞎 女
2. 2. 年齡: ___________歲
3. 身高: _____ 厘米 (或 ____呎____吋)
4. 體重: _______ 公斤 (或_______ 磅)
5. 婚姻狀況: _0_🞎 未婚 _1_🞎 已婚 _2_🞎 分居 / 離婚 _3_🞎 喪偶 _4_🞎 其他 (請列明: ______)
6. 學歷: _0_🞎 小學 _1_🞎中學 _2_🞎大專 _3_🞎 學士或以上
7. 有沒有曾經患上任何嚴重疾病？ _0_🞎 沒有 _1_🞎 有 ☞請列明: _____________
8. 你 現在 有沒有曾經患上任何嚴重疾病？_0_🞎 沒有 _1_🞎 有 ☞請列明: _____________

1. 有沒有曾經做過任何手術？ _0_🞎 沒有 _1_🞎 有 ☞ 什麼手術？ ______________
2. 你現在是否懷孕？ _0_🞎 沒有 _1_🞎 有 _2_🞎 不適用
3. 總括而言，你認為你的健康狀況是: _0_🞎 差 _1_🞎 一般 _2_🞎良好 _3_🞎 很好 _4_🞎 極佳
4. 你有否進行每週 最少3次，每次20分鐘 的中等強度 運動 (過程中會令你輕微流汗) ？

_0_🞎從不 _1_🞎很少 _2_🞎有時 _3_🞎經常

1. 有沒有吸煙習慣？ _0_🞎 非吸煙人士 _1_🞎 吸煙者 _2_🞎 已戒煙人士 ☞ 戒煙年期: ________
2. 有沒有嗜酒習慣？ _0_🞎 非嗜酒人士 _1_🞎 嗜酒人士 _2_🞎 已戒酒人士 ☞ 戒酒年期: ________
3. 有沒有每天做伸展運動 ? _0_🞎 沒有 _1_🞎 有
4. 有沒有每天做肌肉强化運動 ? _0_🞎 沒有 _1_🞎 有
5. 有沒有接受過 搬抬 / 扶抱病人 訓練 ?  _0_🞎 沒有 _1_🞎 有
6. 你現時的職位? _0_🞎 護理員(PCW) _1_🞎 保健員 (HW)
7. 你在現時這個職位工作了多久? _______ 年
8. 一般來說，你須要超時工作嗎? _0_🞎不須要 _1_🞎須要

# **第二部** **肌肉筋骨問題**

**21. 請在圖1及圖2上標示出你的肌肉筋骨問題。**

# 指引：:

# 請 圈出 的現時有問題 (例如痛楚、酸痛、不舒服、麻痺) 的位置的名稱

**圖2**

**圖1**

1. 再在公仔身上劃上交叉「X」以顯示出你出現不適的正確位置


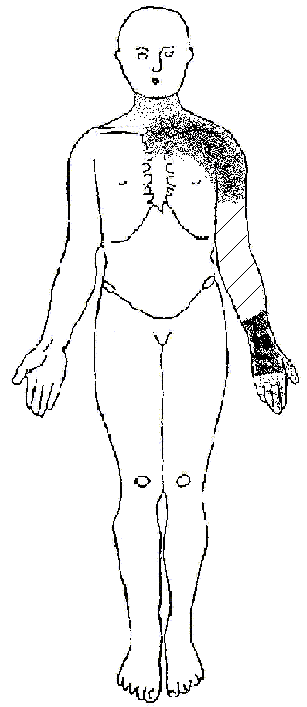

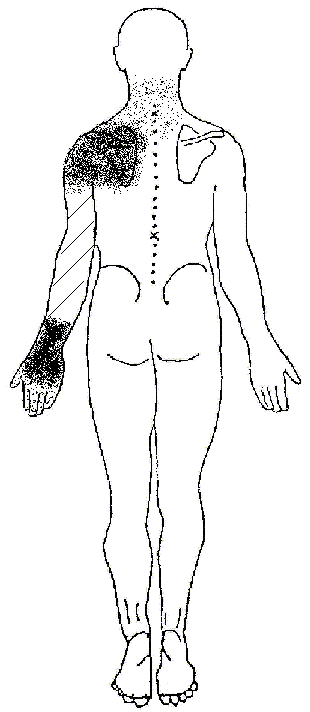


上背

腰背

臀部/大腿

膝蓋

小腿

腳踝 / 腳掌

肩膊

手肘 / 前臂

手掌 / 手腕

手指

頸椎部位


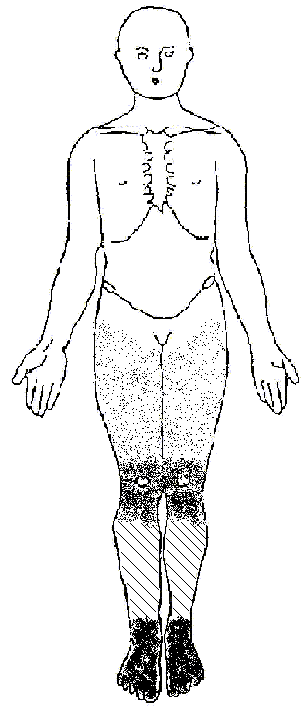

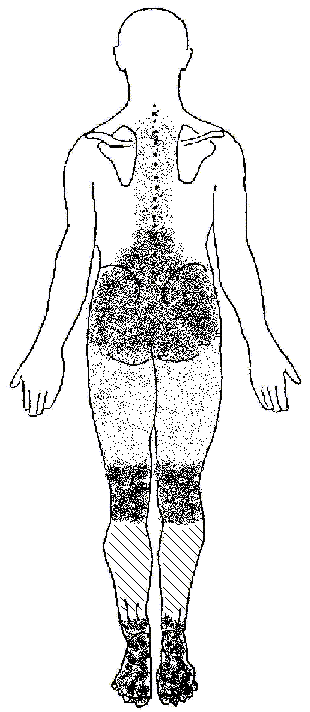


1. **請在適當的方格中打上剔號「✓」。**

|  | 肩膊 |
| --- | --- |
| 1. 你已有此問題多久？ | _0_🞎 <1個月  _1_🞎 1-6個月  _2_🞎 7-11個月  _3_🞎 ≧1 – 3＜年  _4_🞎 ≧3 – 5＜年  _5_🞎 ≧5年 |
| 2. 你認為此問題是否與你的工作有關？ | _0_🞎否  _1_🞎是 |

**第三部 工作情況**

**23.** **請評估你在進行以下日常主要工作的自覺吃力程度。** 您只須選擇**最能形容**您的情況作為答案.

請根據以下的自覺吃力程度指標*來圈出最適當的分數

| 0 | 1 | 2 | 3 | 4 | 5 | 6 | 7 | 8 | 9 | 10 |
| --- | --- | --- | --- | --- | --- | --- | --- | --- | --- | --- |
| 毫無感覺 | 非常微弱 | 微弱 | 普通 | 稍吃力 | 吃力 |  | 很吃力 |  |  | 非常、非常吃力 |

|  | 自覺吃力程度* |
| --- | --- |
| 日常主要工作的種類 | 0 1 2 3 4 5 6 7 8 9 10 |
| 1.把院友由床移至輪椅/椅 (及由輪椅/椅至床) | 0 1 2 3 4 5 6 7 8 9 10 |
| 2.把院友由輪椅移至坐廁 (及由坐廁至輪椅) | 0 1 2 3 4 5 6 7 8 9 10 |
| 3.把院友由浴床/便椅移至床 (及由床至浴床/便椅) | 0 1 2 3 4 5 6 7 8 9 10 |
| 4.重新移正在輪椅上/椅上/床上的院友的姿勢 | 0 1 2 3 4 5 6 7 8 9 10 |
| 5.為院友穿上或脫下衣服 | 0 1 2 3 4 5 6 7 8 9 10 |
| 6.為院友更換尿片 | 0 1 2 3 4 5 6 7 8 9 10 |
| 7.帮院友穿上約束衣/手帶 | 0 1 2 3 4 5 6 7 8 9 10 |
| 8.為院友洗澡 | 0 1 2 3 4 5 6 7 8 9 10 |
| 9.端送餐盤給院友 | 0 1 2 3 4 5 6 7 8 9 10 |
| 10.餵飼臥床院友 | 0 1 2 3 4 5 6 7 8 9 10 |
| 11.為院友整理床舖 (有院友在床) | 0 1 2 3 4 5 6 7 8 9 10 |
| 12.為院友整理床舖 (沒有院友在床) | 0 1 2 3 4 5 6 7 8 9 10 |
| 13.嘗試阻止院友跌倒 | 0 1 2 3 4 5 6 7 8 9 10 |
| 14. 使用人力從地上把院友抬起 | 0 1 2 3 4 5 6 7 8 9 10 |
| 15.移動完全無法自己活動的院友 | 0 1 2 3 4 5 6 7 8 9 10 |
| 16.移動不願意合作的院友 | 0 1 2 3 4 5 6 7 8 9 10 |
| 17.與同事進行搬抬院友的工作時出現不協調 | 0 1 2 3 4 5 6 7 8 9 10 |
| 18.運送衣物往洗衣機/ 乾衣機 | 0 1 2 3 4 5 6 7 8 9 10 |
| 19.為院友晾曬在洗衣機清洗後的衣物 | 0 1 2 3 4 5 6 7 8 9 10 |
| 20.為院友摺疊晾曬好衣物 | 0 1 2 3 4 5 6 7 8 9 10 |
| 21.清潔工作環境 (例如：清潔風扇、抹枱、抹牆身等) | 0 1 2 3 4 5 6 7 8 9 10 |
| 22.推動 損壞或保養差劣的工具 (例如：浴床、輪椅) | 0 1 2 3 4 5 6 7 8 9 10 |
| 23.其他，請註明: ______________________ | 0 1 2 3 4 5 6 7 8 9 10 |

# **第四部 工作處理**

# 24. 您只須選擇**最能形容**您的情況作為答案。

| 下列選項是對工作方式的描述，根據您工作時的情況，選出最適合的選項 | 幾乎從不 | 很少 | 有時 | 經常 | 幾乎總是 |
| --- | --- | --- | --- | --- | --- |
| 1. 我會在疼痛、不舒適的情況下繼續工作，這樣才不會影響我的工作質量。 | _0_⬜ | _1_⬜ | _2_⬜ | _3_⬜ | _4_⬜ |
| 2. 在工作時，我的頸/肩膊/雙手/雙臂/背/臀/大小腿/膝蓋/脚會感到疲勞。 | _0_⬜ | _1_⬜ | _2_⬜ | _3_⬜ | _4_⬜ |
| 3. 在工作時，我會感到疼痛。 | _0_⬜ | _1_⬜ | _2_⬜ | _3_⬜ | _4_⬜ |
| 4. 因為我對自己的頸/上肢/背/下肢痛毫無辦法，所以只能忍痛繼續工作。 | _0_⬜ | _1_⬜ | _2_⬜ | _3_⬜ | _4_⬜ |
| 5. 我實在沒有辦法消除或緩解自己頸/上肢/背/下肢所出現的各種症狀。 | _0_⬜ | _1_⬜ | _2_⬜ | _3_⬜ | _4_⬜ |
| 6. 我的頸/上肢/背/下肢（其中一處或多處）會做一些急促、猛烈、快速、突然的動作。 | _0_⬜ | _1_⬜ | _2_⬜ | _3_⬜ | _4_⬜ |
| 7. 我不能中途停工，因為這樣做會讓其他同事對我有意見。 | _0_⬜ | _1_⬜ | _2_⬜ | _3_⬜ | _4_⬜ |
| 8. 我不能中途停工，因為這樣做會讓上司失望或增加他的負擔。 | _0_⬜ | _1_⬜ | _2_⬜ | _3_⬜ | _4_⬜ |
| 9. 我不能中途停工，因為這樣做會讓同事失望或增加他們的負擔 | _0_⬜ | _1_⬜ | _2_⬜ | _3_⬜ | _4_⬜ |
| 10. 我不能中途停工，因為這樣做會影響我的評估、晉升，和/或讓我丟掉工作。 | _0_⬜ | _1_⬜ | _2_⬜ | _3_⬜ | _4_⬜ |
| 11. 如果我關心自己的健康而放下工作，放鬆一下或做做運動，我的同事/上司會對我有意見。 | _0_⬜ | _1_⬜ | _2_⬜ | _3_⬜ | _4_⬜ |
| 12. 儘管我在工作中付出了很大努力，但我還是真的不知道我的工作是否得到應有的認同。 | _0_⬜ | _1_⬜ | _2_⬜ | _3_⬜ | _4_⬜ |
| 13. 如果我沒完成自己的工作，上司不會讓我好過。 | _0_⬜ | _1_⬜ | _2_⬜ | _3_⬜ | _4_⬜ |
| 14. 如果我向主管反映（一些）問題，比如某某同事沒有努力做好自己的本職工作，這根本起不了什麼作用，所以不如自己做多點。 | _0_⬜ | _1_⬜ | _2_⬜ | _3_⬜ | _4_⬜ |
| 15. 上司或其他人對工作質量的要求與我不同，這一點讓我感到很沮喪。 | _0_⬜ | _1_⬜ | _2_⬜ | _3_⬜ | _4_⬜ |
| 16. 我的工作有太多的最後期限，所以工作總是做不完。 | _0_⬜ | _1_⬜ | _2_⬜ | _3_⬜ | _4_⬜ |
| 17. 儘管我會有條理地安排自己的工作，以便能夠在最後期限前完成工作，但情況在不斷變化，自己還是要更加努力地工作，以便按時完成。 | _0_⬜ | _1_⬜ | _2_⬜ | _3_⬜ | _4_⬜ |
| 18. 我的工作時間表很難控制。 | _0_⬜ | _1_⬜ | _2_⬜ | _3_⬜ | _4_⬜ |
| 19. 我在工作時會感到壓力。 | _0_⬜ | _1_⬜ | _2_⬜ | _3_⬜ | _4_⬜ |
| 20. 工作中我會敦促自己，確立自己的表現高於上司和其他人的預期目標。 | _0_⬜ | _1_⬜ | _2_⬜ | _3_⬜ | _4_⬜ |
| 21. 我的同事沒有做好自己的份內工作，我就得承擔更多的工作。 | _0_⬜ | _1_⬜ | _2_⬜ | _3_⬜ | _4_⬜ |
| 22. 別人會告訴我應該放慢節奏，工作不要那麼拼命。 | _0_⬜ | _1_⬜ | _2_⬜ | _3_⬜ | _4_⬜ |
| 23. 在日常工作期間，我會中途停下來休息，做做伸展運動。 | _0_⬜ | _1_⬜ | _2_⬜ | _3_⬜ | _4_⬜ |
| 24. 在工作時，我會不時停下來休息。 | _0_⬜ | _1_⬜ | _2_⬜ | _3_⬜ | _4_⬜ |

# **第五部 有關體力處理操作**

| **25. 請圈上你認為正確的答案** | |  |  |
| --- | --- | --- | --- |
| 1. | 當搬抬依賴性高的院友時，如果你的同事未能協助你搬抬院友，你自己一人搬抬院友是可接受和合適的 | 對 | 錯 |
|  | 彎曲腰部照顧院友不是導致肌肉筋骨勞損的其中一個風險因素 | 對 | 錯 |
|  | 當搬抬院友或其他物件時， 你應該屈曲你的膝部 (而不是腰部) ，並用雙腳 (而不是背部) 去做提舉的動作 | 對 | 錯 |
|  | 在搬抬院友或其他物件時，你應該要盡量站遠你將會提起的人/物件 | 對 | 錯 |
|  | 你應該要企在院友的較有力的一側協助轉移院友 | 對 | 錯 |
|  | 找時間來停一停或伸展筋骨是一個好的習慣 | 對 | 錯 |
|  | 減低肌肉筋骨勞損風險的主要方法是避免使用有危害性的人力操作 | 對 | 錯 |
|  | 過份用力及重複性的動作是引致肌肉筋骨勞損的其中誘因 | 對 | 錯 |
|  | 長時間俯身是不良的姿勢，可引致肌肉筋骨勞損 | 對 | 錯 |
|  | 大幅度的側身扭動是不良姿勢 | 對 | 錯 |
|  | 突然急促發力拉動橫單轉移院友會增加受傷風險 | 對 | 錯 |
|  | 推動設備損壞或保養差劣的搬運工具(例如：輪椅、浴床) 可引致肌肉筋骨勞損 | 對 | 錯 |
|  | 利用推床、輪椅、淋浴/便桶椅、或機械搬抬設施，可消除或減少搬抬的氣力或負擔。 | 對 | 錯 |
|  | 換床單時，理想的工作高度是在你的腰部水平。 | 對 | 錯 |
|  | 把床的高度調節至輪椅的高度，你便可將院友平穩地橫向移送而減少抬起院友。 | 對 | 錯 |
|  | 在將進行搬抬/移動前，你不需要向院友解釋清楚因為院友不會明白。 | 對 | 錯 |
|  | 在你的臀部至胸部之間的位置進行搬抬動作最有力。 | 對 | 錯 |
|  | 推入/拉出較左/右移動(橫過身體) 有力。 | 對 | 錯 |
|  | 放工回家後坐在沙發/躺在床上不動是最好舒緩肌肉筋骨疲勞的方法。 | 對 | 錯 |
|  | 伸展運動一定須要一氣呵成從頭到脚做。 | 對 | 錯 |
|  | 返工前後及返工時都須要做伸展運動以減少肌肉筋骨勞損。 | 對 | 錯 |

# **第六部 人體學之風險**

26. 你認為以下那一項描述是**導致**你的**肌肉筋骨問題**的**主**要原**因**呢？請評估你有以下情况的**頻密程度**

|  | **是/否** | **從不 間中 較多 經常** |
| --- | --- | --- |
| 1. 上身或上肢維持固定或不良的姿勢 (如向前俯身彎腰、向側面彎腰、大幅度側身扭動、屈曲身體、彎曲或扭動脊椎；手伸高至肩以上的高度、雙臂過度伸展如越過床欄、長時間維持一種姿勢等) | _0_🞎 否 _1_🞎 是 | 0 1 2 3 |
| 2. 上身或上肢做出重複性的動作 (如搬移院友、執拾整理院友的空間、換尿片等) | _0_🞎 否 _1_🞎 是 | 0 1 2 3 |
| 3. 下身或下肢維持固定或不良的姿勢(如長時間站立、蹲坐、跪下、彎曲膝蓋、長時間維持一種姿勢等) | _0_🞎 否 _1_🞎 是 | 0 1 2 3 |
| 4. 下身或下肢做出重複性的動作(如快速走動等) | _0_🞎 否 _1_🞎 是 | 0 1 2 3 |
| 5. 使勁地使用一些工具 (如勁力地抓握沒有把手的橫或被單作院友轉移) | _0_🞎 否 _1_🞎 是 | 0 1 2 3 |
| 6. 不恰當的傢俬(如床、輪椅、檯子)安排 (如不能調校床的高度﹑床邊靠牆、非電動床、沒有起人機、起人機較大不方便在狹小的病房搬移院友等) | _0_🞎 否 _1_🞎 是 | 0 1 2 3 |
| 7. 使用損壞或保養差劣的工具 (例如：浴床、輪椅、起人機等) | _0_🞎 否 _1_🞎 是 | 0 1 2 3 |
| 8. 工作環境內的環境因素 (如燈光、溫度、梯級等) | _0_🞎 否 _1_🞎 是 | 0 1 2 3 |
| 9. 工作壓力大或不滿意現在所從事的工作 | _0_🞎 否 _1_🞎 是 | 0 1 2 3 |

# **第七部 一般工作情況**

28. 請把**最能形容**您對下列句子的同意或不同意的程度，**在適當方格內打上「√」號**：

|  | **非常不同意**  **(1)** | **不同意**  **(2)** | **同意**  **(3)** | **非常同意**  **(4)** |
| --- | --- | --- | --- | --- |
| 1. 我的工作要求我學習新事物。 | _1_🞎 | _2_🞎 | _3_🞎 | _4_🞎 |
| 2. 我的工作涉及很多重複的工序。 | _1_🞎 | _2_🞎 | _3_🞎 | _4_🞎 |
| 3. 我的工作要求我具備創意。 | _1_🞎 | _2_🞎 | _3_🞎 | _4_🞎 |
| 4. 我的工作要求我有高度技巧。 | _1_🞎 | _2_🞎 | _3_🞎 | _4_🞎 |
| 5.我需在工作上處理不同種類的事務。 | _1_🞎 | _2_🞎 | _3_🞎 | _4_🞎 |
| 6. 我有機會發展個人的特別技能。 | _1_🞎 | _2_🞎 | _3_🞎 | _4_🞎 |
| 7. 我的工作容許我有很大的決策權。 | _1_🞎 | _2_🞎 | _3_🞎 | _4_🞎 |
| 8. 在我的工作中，我有很少自由決定如何做我的工作。 | _1_🞎 | _2_🞎 | _3_🞎 | _4_🞎 |
| 9. 對於我的工作情況我有很大發言權。 | _1_🞎 | _2_🞎 | _3_🞎 | _4_🞎 |
| 10. 我的工作要求我做事很迅速。 | _1_🞎 | _2_🞎 | _3_🞎 | _4_🞎 |
| 11. 我的工作要求我做事很勤奮。 | _1_🞎 | _2_🞎 | _3_🞎 | _4_🞎 |
| 12. 我沒有被要求做過量的工作。 | _1_🞎 | _2_🞎 | _3_🞎 | _4_🞎 |
| 13. 我有充足時間完成工作。 | _1_🞎 | _2_🞎 | _3_🞎 | _4_🞎 |
| 14. 我沒有遇上其他人對我作出互相矛盾的要求。 | _1_🞎 | _2_🞎 | _3_🞎 | _4_🞎 |
| 15. 我的工作要求長時間對工序高度集中。 | _1_🞎 | _2_🞎 | _3_🞎 | _4_🞎 |
| 16. 我的工序常在完成之前被打斷，而需在稍後時間再度專注。 | _1_🞎 | _2_🞎 | _3_🞎 | _4_🞎 |
| 17. 我的工作是非常忙亂的。 | _1_🞎 | _2_🞎 | _3_🞎 | _4_🞎 |
| 18. 等候其他人或其他部門工作經常拖慢我的進度。 | _1_🞎 | _2_🞎 | _3_🞎 | _4_🞎 |
| 19. 我的上司關注他/她下屬的福利。 | _1_🞎 | _2_🞎 | _3_🞎 | _4_🞎 |
| 20. 我的上司留意我所講的說話。 | _1_🞎 | _2_🞎 | _3_🞎 | _4_🞎 |
| 21. 我的上司對我有敵意而且工作上有衝突。 | _1_🞎 | _2_🞎 | _3_🞎 | _4_🞎 |
| 22. 我的上司在我完成工作方面很幫得上忙。 | _1_🞎 | _2_🞎 | _3_🞎 | _4_🞎 |
| 23. 我的上司在維繫各人合作方面很成功。 | _1_🞎 | _2_🞎 | _3_🞎 | _4_🞎 |
| 24. 和我工作的人有足夠的能力勝任他們的工作。 | _1_🞎 | _2_🞎 | _3_🞎 | _4_🞎 |
| 25. 和我工作的人很關心我個人的情況。 | _1_🞎 | _2_🞎 | _3_🞎 | _4_🞎 |
| 26. 和我工作的人對我有敵意而且工作上有衝突。 | _1_🞎 | _2_🞎 | _3_🞎 | _4_🞎 |
| 27. 和我工作的人都友善。 | _1_🞎 | _2_🞎 | _3_🞎 | _4_🞎 |
| 28. 和我工作的人互相鼓勵合力工作。 | _1_🞎 | _2_🞎 | _3_🞎 | _4_🞎 |
| 29. 和我工作的人在我完成工作方面很幫得上忙。 | _1_🞎 | _2_🞎 | _3_🞎 | _4_🞎 |
| 30. 院友與他們的家人很關心我個人的情況。 | _1_🞎 | _2_🞎 | _3_🞎 | _4_🞎 |
|  | **非常不同意**  **(1)** | **不同意**  **(2)** | **同意**  **(3)** | **非常同意**  **(4)** |
| 31. 院友與他們的家人對我有敵意而且工作上有衝突。 | _1_🞎 | _2_🞎 | _3_🞎 | _4_🞎 |
| 32. 院友與他們的家人都友善。 | _1_🞎 | _2_🞎 | _3_🞎 | _4_🞎 |
| 33. 院友與他們的家人都互相鼓勵合力工作。 | _1_🞎 | _2_🞎 | _3_🞎 | _4_🞎 |
| 34. 院友與他們的家人在我完成工作方面很幫得上忙。 | _1_🞎 | _2_🞎 | _3_🞎 | _4_🞎 |
| 35. 我的工作要求我走路走得很快。 | _1_🞎 | _2_🞎 | _3_🞎 | _4_🞎 |
| 36. 我的工作要求大量體力勞動。 | _1_🞎 | _2_🞎 | _3_🞎 | _4_🞎 |
| 37. 我常在工作時被要求移動或抬起很重的物件。 | _1_🞎 | _2_🞎 | _3_🞎 | _4_🞎 |
| 38. 我的工作要求非常迅速和持續的身體活動。 | _1_🞎 | _2_🞎 | _3_🞎 | _4_🞎 |
| 39. 我常要在上身或上肢維持彆扭的姿勢下長時間工作。 | _1_🞎 | _2_🞎 | _3_🞎 | _4_🞎 |
| 40. 我常要在下身或下肢維持彆扭的姿勢下長時間工作。 | _1_🞎 | _2_🞎 | _3_🞎 | _4_🞎 |

**第八部 其他工作情況**

**29, 請在空格內劃上以表達你對現職工作的看法。**

| 1. | 我對現時的工作感到滿意 | □ 十分滿意 | □ 滿意 | □ 不滿意 | □ 十分不滿意 |
| --- | --- | --- | --- | --- | --- |
| 2. | 我對現時的工作感到壓力 | □十分有壓力 | □ 有壓力 | □ 沒有壓力 | □十分沒有壓力 |
| 3. | 我想過要申請離職 | □ 有想過 | | □ 沒有想過 | |

| 4. 你認為以自己的健康情況能否在現職繼續工作兩年？ _1_ 不可能 _2_ 不肯定 _3_ 相當肯定 |
| --- |

******************************************************************************************

## 多謝你寶貴的時間以完成這份問卷
